# Role of TRPV1 Channels in Ischemia/Reperfusion-Induced Acute Kidney Injury

**DOI:** 10.1371/journal.pone.0109842

**Published:** 2014-10-17

**Authors:** Lan Chen, Lajos Markó, Mario Kaßmann, Ye Zhu, Kaiyin Wu, Maik Gollasch

**Affiliations:** 1 Experimental and Clinical Research Center (ECRC), a joint cooperation between the Charité Medical Faculty and the Max Delbrück Center for Molecular Medicine (MDC), Lindenberger Weg 80, Berlin, Germany; 2 Xiamen Zhongshan Hospital, Xiamen University, Xiamen, Fujian Province, China; 3 Institute of Pathology, Charité Campus Mitte, Charitéplatz 1, Berlin, Germany; 4 Medical Clinic for Nephrology and Internal Intensive Care, Charité Campus Virchow, Augustenburger Platz 1, Berlin, Germany; Duke University Medical Center, United States of America

## Abstract

**Objectives:**

Transient receptor potential vanilloid 1 (TRPV1) -positive sensory nerves are widely distributed in the kidney, suggesting that TRPV1-mediated action may participate in the regulation of renal function under pathophysiological conditions. Stimulation of TRPV1 channels protects against ischemia/reperfusion (I/R)-induced acute kidney injury (AKI). However, it is unknown whether inhibition of these channels is detrimental in AKI or not. We tested the role of TRPV1 channels in I/R-induced AKI by modulating these channels with capsaicin (TRPV1 agonist), capsazepine (TRPV1 antagonist) and using *Trpv1−/−* mice.

**Methods and Results:**

Anesthetized C57BL/6 mice were subjected to 25 min of renal ischemia and 24 hrs of reperfusion. Mice were pretreated with capsaicin (0.3 mg/kg body weight) or capsazepine (50 mg/kg body weight). Capsaicin ameliorated the outcome of AKI, as measured by serum creatinine levels, tubular damage,neutrophil gelatinase-associated lipocalin (NGAL) abundance and Ly-6B.2 positive polymorphonuclear inflammatory cells in injured kidneys. Neither capsazepine nor deficiency of TRPV1 did deteriorate renal function or histology after AKI. Measurements of endovanilloids in kidney tissue indicate that 20-hydroxyeicosatetraeonic acid (20-HETE) or epoxyeicosatrienoic acids (EETs) are unlikely involved in the beneficial effects of capsaicin on I/R-induced AKI.

**Conclusions:**

Activation of TRPV1 channels ameliorates I/R-induced AKI, but inhibition of these channels does not affect the outcome of AKI. Our results may have clinical implications for long-term safety of renal denervation to treat resistant hypertension in man, with respect to the function of primary sensory nerves in the response of the kidney to ischemic stimuli.

## Introduction

The kidneys are profusely innervated organs in which renal adrenergic neurons supply all the segments of renal vasculature and are distributed throughout the renal cortex, outer stripe of the medulla, juxtamedullary region of the inner cortex and the renal tubules [1]. Transient receptor potential cation channel subfamily V member 1 (TRPV1)-positive sensory nerves are widely distributed in the kidney, suggesting that TRPV1-mediated action may participate in the regulation of renal function under pathophysiological conditions [2]. Indeed, sodium excretion in response to sodium loading is impaired in salt-sensitive hypertension induced by surgical sensory denervation or by sensory nerve degeneration following capsaicin treatment [3]. Blockade of TRPV1 increases blood pressure in Wistar or Dahl salt-resistant rats fed a high but not normal salt diet suggesting that high salt intake may activate TRPV1 conferring a protective effect [4]. Chronic TRPV1 stimulation by systemic application of capsaicin lowers systemic blood pressure with possible involvement of TRPV1 channels in sensory nerves and blood vessels [5].

Catheter-based renal denervation (RDN) is a novel technique to lower resistant hypertension specifically targeting renal sympathetic nerves [6]. This procedure is expected to affect the function of TRPV1-rich primary sensory nerves of the renal sympathetic nervous system. Of note, renal sympathetic nerves and circulating catecholamines are believed to be involved in the development of the progressive renal tissue injury accompanying ischemic acute renal failure [7] [8]. However, experimental evidence concerning primary sensory nerves of renal sympathetic nervous system and its contribution to the pathogenesis of ischemia/reperfusion (I/R)-induced acute kidney injury (AKI) is confounding and warrants further investigation.

Three studies have reported that systemic activation of TRPV1 by capsaicin ameliorates I/R-induced renal dysfunction [9] [10] [11]. Capsazepine is a potent blocker of TRPV1 channels [12] [13], but this drug has not been studied in AKI. Deficiency of TRPV1 genes in mice has been shown to impair the recovery process of I/R induced cardiac dysfunction [14]. It is unknown whether inhibition of TRPV1 channels is detrimental in AKI or not. Knowledge of these effects may have clinical implications to understand the kidneys' ability to respond to ischemic stimuli after RDN therapy in man, including the long-term safety profile of this procedure.

In this study, we tested the role of TRPV1 channels in experimental I/R induced AKI in mice by stimulating and inhibiting these channels with capsaicin, capsazepine, respectively, and using *Trpv1−/−* mice. In order to provide possible mechanistic insights, we measured endovanilloids, such as 20-hydroxyeicosatetraeonic acid (20-HETE), anandamide, epoxyeicosatrienoic acids and epoxy-9Z-octadecenoic acids in injured kidneys, which can activate TRPV(1) channels [15] [16] [17] [18] [19] and are implicated in AKI [20] [21] [22] [23]. Our results support the view that activation of TRPV1 channels ameliorates I/R–induced AKI. In contrast to our expectations, we found that inhibition or genetic ablation of TRPV1 does not affect the outcome of AKI. Our findings provide novel insights into the role of TRPV1-rich primary sensory nerves in AKI and have implications for safety of renal denervation in man.

## Materials and Methods

### Animals

Experiments were performed using 2–3-month-old male C57BL/6 wild-type mice and *Trpv1*−/− mice (Jackson Laboratories, Bar Harbor, Main, USA). Mice were housed under a 12 hr/12 hr day/night cycle with free access to food and water. The investigation was approved by the local government authorities and conforms to the Guide for the Care and Use of Laboratory Animals published by the US National Institutes of Health and the ethics policies of our University and the Land Berlin. The protocol was approved by the Committee on the Ethics of Animal Experiments of the Charité and Land Berlin (Permit Number: 0065/11).

### Animal model of renal ischemia/reperfusion

We performed ischemia/reperfusion (I/R)-induced acute kidney injury (AKI) similarly as previously described for rats [22]. Briefly, anaesthesia was performed with isoflurane (2.5%) in air (350 ml/min). A temperature controller with heating pad (TCAT-2, Physitemp Instruments Inc, Clifton, NJ, USA) was used to keep the animal’s body temperature stable during surgery. Rectal body temperature was continuously monitored using a sensor based on thermistors. During surgery, rectal body temperature values (max-min) were as follows: I/R control, 37.8–35.8°C, n = 8; capsaicin (0.3 mg/kg) treatment I/R, 37.8–35.4°C, n = 13; capsazepine (50 mg/kg) treatment I/R, 37.6–35.1°C, n = 10; Trpv1−/− I/R, 37.5–35.6°C, n = 13. I/R injury was induced after right uninephrectomy by clipping the pedicles of the remaining left kidney for 25 minutes with non-traumatic aneurysm clip (FE690K, Aesculap AG, Tuttlingen, Germany). Reperfusion was confirmed visually. After surgery mice had free access to water and chow. Perioperative hydration by applying body-warm sterile physiological saline solution and analgesia by subcutaneous buprenorphin (0.1 mg/kg) was provided for each mouse. Three groups of mice were intraperitoneally injected either with saline (control group), with the capsaicin (0.3 mg/kg body weight) or capsazepine (50 mg/kg body weight) 30 min before I/R injury. After 24 hour reperfusion time the mice were sacrificed, and the kidney and blood was collected for further analysis.

Collected blood was centrifuged at 2,000 g for 10 min for examination of serum creatinine. Serum levels of creatinine were measured by an autoanalyzer (Beckman Analyzer; Beckman Instruments, Munich, Germany). The kidneys were immersed in 4% buffered formalin for histopathological and immunohistochemical examination. In total 51 mice underwent surgery; two of them died during the perioperative period and were excluded from the study.

### Histological evaluation of the kidneys

All kidney samples were immersed in 4% buffered formalin. Samples were processed for histology in an automatic device. Processing consisted of three immersions (1 h each) in absolute ethyl alcohol and three immersions (1 h each) in xylene at room temperature, followed by immersion in two vessels (1 h in each) containing liquid paraffin at approximately 60°C.

After embedding, the samples were blocked in paraffin and cut into 1.5-µm sections using a microtome. Sections were mounted on slides, deparaffinized in xylene and stained with hematoxylin and eosin (H&E), periodic acid-Schiff (PAS).

Histomorphometric analysis was performed to semi-quantitively evaluate the score of acute tubular necrosis (ATN) in mouse renal parenchyma using H&E staining. Slides were examined under a light microscope (200X magnification). Examination was performed by a local nephropathologist, who was blinded to the experimental assignments. Acute tubular injury (ATI) was observed in this study to assess the reversible tubular damage due to ischemia, the histologic features of ATI included one or more of the following lesions: tubular epithelial swelling with lucency of the cytoplasm, loss of brush border, luminal dilatation with simplification of the epithelium, and cytoplasmic vacuolization. Acute tubular necrosis (ATN) was also evaluated, which was indicated by patchy loss of tubular epithelial cells with resultant gaps and exposure of denuded basement membrane, evidence of cellular regeneration, as well as obliterated tubular hyaline and/or granular casts. The histological findings were graded from 0 to 3 according to the distribution of lesions: 0 = none; 1 = <25%; 2 = 25–50%; 3 = >50% [24].

### Neutrophil gelatinase-associated lipocalin (NGAL) immunohistochemistry

NGAL immunohistochemistry was performed on paraffin-fixed kidney sections. Non-specific binding sites were blocked with 10% normal donkey serum for 30 min. Then sections were incubated with the NGAL antibody (dilution: 1∶300) overnight at 4°C in a humid chamber. For fluorescence visualization of bound primary antibody, sections were further incubated with Cy3-conjugated secondary antibody for 1 h in a humid chamber at room temperature [25]. Specimens were analyzed using a Zeiss Axioplan-2 imaging microscope with the computer program AxioVision 4.8 (Zeiss, Jena, Germany). The investigator had no knowledge of the treatment group assignment. NGAL positive area were analysed by ImageJ (NIH, USA) using thresholding method.

### Immunohistochemical detection of Ly-6B.2 positive cells

Immunohistochemical staining of Ly-6B.2 positive cells was performed on paraffin-fixed kidney sections. Antigen retrieval was performed by incubating sections for 10 minutes at 37°C in a trypsin solution (Sigma). After cooling down non-specific binding sites were blocked with 10% normal donkey serum for 30 min. Then sections were incubated with the rat anti mouse Ly-6B.2 monoclonal antibody, clone 7/4 (dilution: 1∶300) overnight at 4°C in a humid chamber. For fluorescence visualization of bound primary antibody, sections were further incubated with Cy3-conjugated secondary antibody for 1 h in a humid chamber at room temperature [17]. Specimens were analyzed using a Zeiss Axioplan-2 imaging microscope with the computer program AxioVision 4.8 (Zeiss, Jena, Germany). The investigator had no knowledge of the treatment group assignment. Ly-6B.2 positive cells were counted in per field view by a counter. (400X magnification).

### Determination of endovanilloids

Endovanilloid profiles were measured by Lipidomics GmbH (Berlin, Germany). Briefly, kidney tissue samples were subjected to alkaline hydrolysis and subsequent solid phase extraction was performed as described previously [26] [27]. LC-MS/MS analysis of the extracted metabolites was performed using the Agilent 6460 Triplequad mass spectrometer with Jetstream ion source (Agilent Technolgies, Santa Clara, CA) coupled with Agilent 1200 HPLC (degasser, binary pump, well plate sampler, thermostated column compartment). The HPLC system was equipped with a Phenomenex Kinetex Column (150 mm×2.1 mm, 2.6 µm, Phenomenex, Aschaffenburg, Germany). Tissue samples were analysed in two separate series of experiments, each using independent internal standards (deuterated eicosanoids and metabolites) added to the samples before extraction for the quantification of groups of similar metabolites. The first series of experiments determined eicosanoid profiles in non-ischemic (Control) and ischemic (I) kidneys 25 min after ischemia (without reperfusion) from a group of wild-type mice with ischemia (I)-induced AKI; the second series of experiments determined the profiles in non-ischemic (I/R Control) and ischemic (I/R) kidneys after 25 min of ischemia and 24 hours of reperfusion from the above described four groups of mice with I/R-induced AKI, namely control wild-type mice, capsaicin treated wild-type mice, capsazepine treated wild-type mice, and *Trpv1*−/− mice.

### Materials

Capsaicin and capsazepine were obtained from Sigma-Aldrich Chemie GmbH (Munich, Germany). NGAL antibody was obtained from R&D systems (AF1857). Rat anti Mouse Ly-6B.2 monoclonal antibody, clone 7/4 was obtained from AbD Serotec (Puchheim, Germany). Cy3-conjugated secondary antibody was obtained from Jackson ImmunoResearch (West Grove, USA).

### Statistics

All data are given as mean ± SEM of n experiments. Statistical analysis of data was performed using one-way ANOVA using ORIGIN 6.0 software. A *P* value of less than 0.05 was considered statistically significant.

## Results

### Serum creatinine levels after I/R-induced AKI

**Figure 1:**
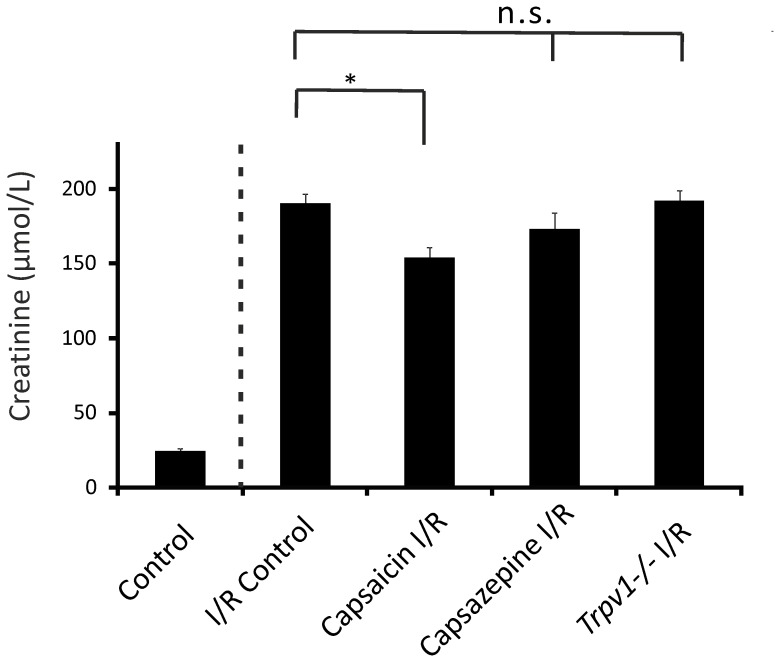
Serum creatinine levels in mice subjected to renal I/R injury were ameliorated by pretreatment with capsaicin, but not capsazepine or genetic ablation of TRPV1. Control, control mice that underwent sham surgery (nephrectomy) without I/R. I/R Control, control mice that underwent renal I/R injury. Capsaicin I/R, wild-type mice pretreated with capsaicin followed by renal I/R injury. Capsazepine I/R, wild-type mice pretreated with capsazepine followed by renal I/R injury. *Trpv1−/−* mice that underwent renal I/R injury. *P<0.05. *P*>0.05; not significant.

Mice treated with capsaicin (0.3 mg/kg) had lower serum creatinine levels after I/R-induced AKI compared to control mice after renal I/R injury (Figure 1, 154.35±6.58 µmol/L, n = 13, vs 190.84±6.13 µmol/L, n = 8, respectively, *P*<0.05). However, mice treated with capsazepine (50 mg/kg; Figure 1, 173.51±10.78 µmol/L, n = 10, *P*>0.05) showed serum creatinine levels after AKI similar to control mice after renal I/R injury. *Trpv1−/−* mice showed an increase in serum creatinine levels after I/R induced AKI (Figure 1, 192.51±6.76 µmol/L, n = 13, *P*>0.05) similar to control mice after renal I/R injury.

### Tubular damage scores

**Figure 2:**
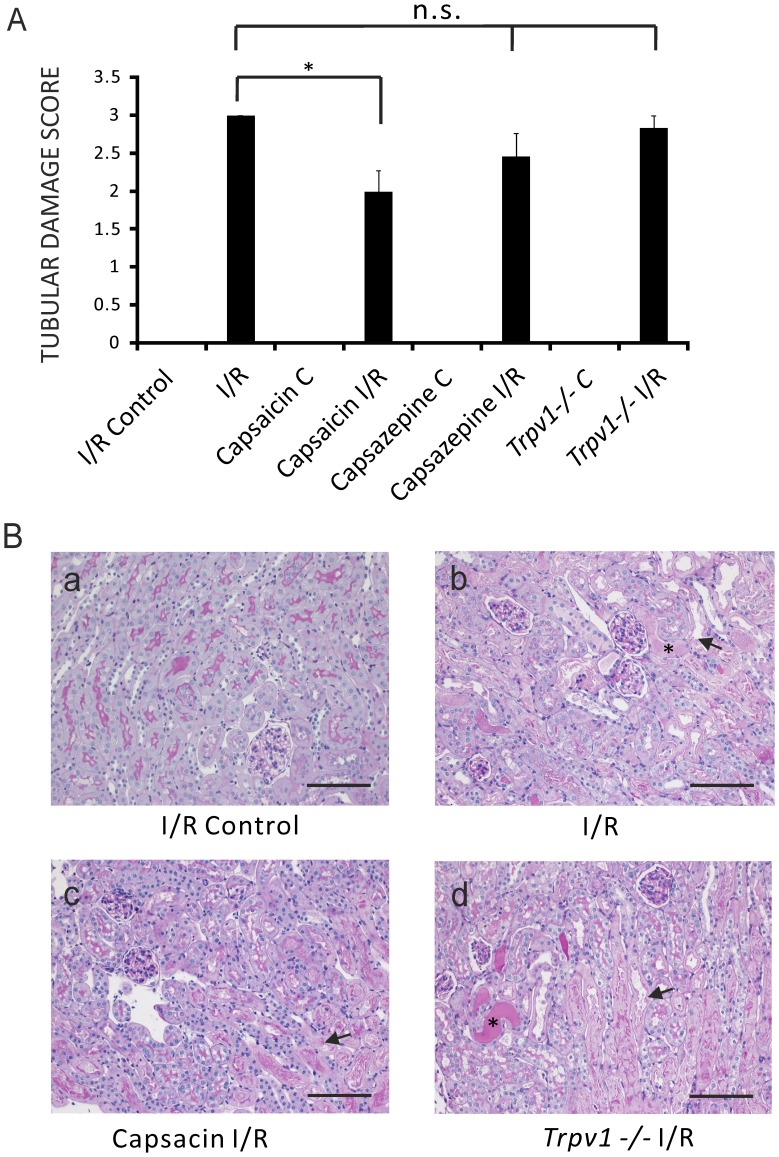
Amelioration of tubular damage score of I/R-induced AKI by pretreatment of mice with capsaicin, but not with capsazepine or genetic ablation of TRPV1. **Panel A**: Tubular damage scores of ipsilateral kidneys (I/R Control) and kidneys after I/R (I/R) of control mice. Tubular damage scores of ipsilateral kidneys (Capsaicin C) and kidneys after I/R injury (Capsaicin I/R) of mice pretreated with capsaicin. Tubular damage scores of ipsilateral kidneys (Capsazepine C) and kidneys after I/R injury (Capsazepine I/R) of mice pretreated with capsazepine. Tubular damage scores of ipsilateral kidneys (*Trpv1−/−* C) and kidneys after I/R injury (*Trpv1−/−* I/R) of *Trpv1−/−* mice. Scores of ipsilateral control kidneys without I/R injury were all 0. *, *P*<0.05. *P*≥0.05; not significant. **Panel B:** Histological sections. (**a**) I/R Control, histology of ipsilateral kidney before renal I/R injury of a control mouse. (**b**) I/R, histology of kidney after I/R injury of a control mouse. The section shows acute tubular necrosis characterized by loss of tubular epithelial cells (arrow) and shedding of the brush border (asterisk). (**c**) Capsaicin I/R, histology of kidney after I/R injury of a mouse pretreated with capsaicin. Acute tubular necrosis (arrow). (**d**) *Trpv1−/−* I/R, histology of kidney after I/R injury of a *Trpv1−/−* mouse. Acute tubular necrosis (arrow) and shedding of the brush border (asterisk). Scale bar 100 µm. Magnification × 200.

Capsaicin (0.3 mg/kg) ameliorated I/R-induced tubular damage compared to control mice after renal I/R injury (Figure 2A, ATN scores 2.0±0.27, n = 13, vs 3.0±0, n = 8, respectively, *P*<0.05). ATN score in mice treated with capsazepine (50 mg/kg) (Figure 2A, ATN 2.45±0.31, n = 10, *P*>0.05) was not different compared to control mice after renal I/R injury. *Trpv1−/−* mice after AKI showed a tubular damage score similar to control mice after renal I/R injury (Figure 2A, ATN 2.83±0.17, n = 13, *P*>0.05).

### NGAL abundance in the kidney after AKI

**Figure 3:**
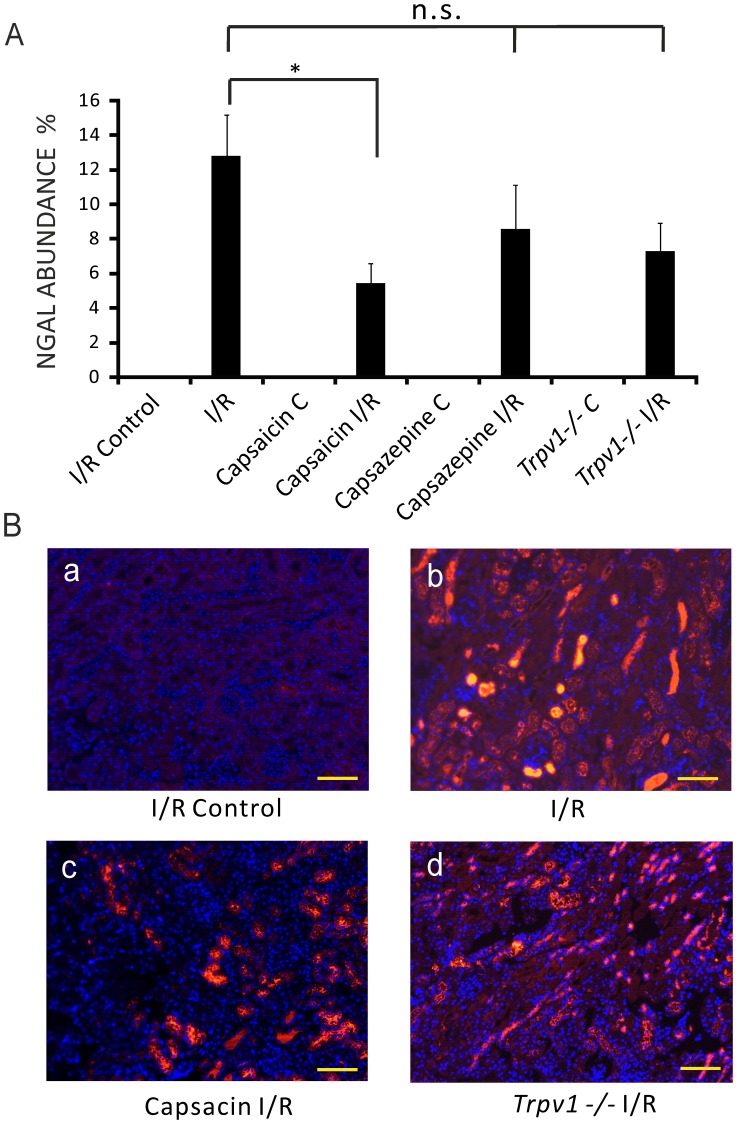
NGAL abundance in the kidney was lower in I/R-induced AKI in mice treated with capsaicin, but not with capsazepine or in *Trpv1−/−* mice. **Panels A:** NGAL abundance of ipsilateral kidneys (I/R Control) and kidneys after I/R (I/R) injury of control mice. NGAL abundance of ipsilateral kidneys (Capsaicin C) and kidneys after I/R injury (Capsaicin I/R) of mice pretreated with capsaicin. NGAL abundance of ipsilateral kidneys (Capsazepine C) and kidneys after I/R injury (Capsazepine I/R) of mice treated with capsazepine. NGAL abundance of ipsilateral kidneys (*Trpv1*−/− C) and kidneys after I/R injury (*Trpv1−/−* I/R) of *Trpv1−/−* mice. *, *P*<0.05. *P*>0.05; not significant. **Panels B:** Immunohistological sections. (**a**) I/R Control, images of NGAL negative staining of ipsilateral kidney before renal I/R injury of a control mouse. (**b**) I/R, intense renal NGAL staining after I/R injury of a control mouse. (**c**) Capsaicin I/R, renal NGAL staining after I/R injury of a mouse pretreated with capsaicin. (**d**) *Trpv1−/−* I/R, renal NGAL staining after I/R injury of a *Trpv1−/−* mouse. Scale bar 100 µm. Magnification ×200.

Mice treated with capsaicin (0.3 mg/kg) showed lower NGAL abundance, an early biomarker of AKI, in kidneys after I/R injury compared to control mice after AKI (Figure 3A, 5.44±1.13%, n = 13, vs 12.78±2.40%, n = 8, respectively, *P*<0.05). Kidneys of mice treated with capsazepine (50 mg/kg, Figure 3A, 8.56±2.57%, n = 10, *P*>0.05) or *Trpv1−/−* mice (Figure 3A, 7.25±1.67%, n = 13, *P*>0.05) showed NGAL abundance similar to control mice after I/R injury.

### Ly-6B.2 positive cells

**Figure 4:**
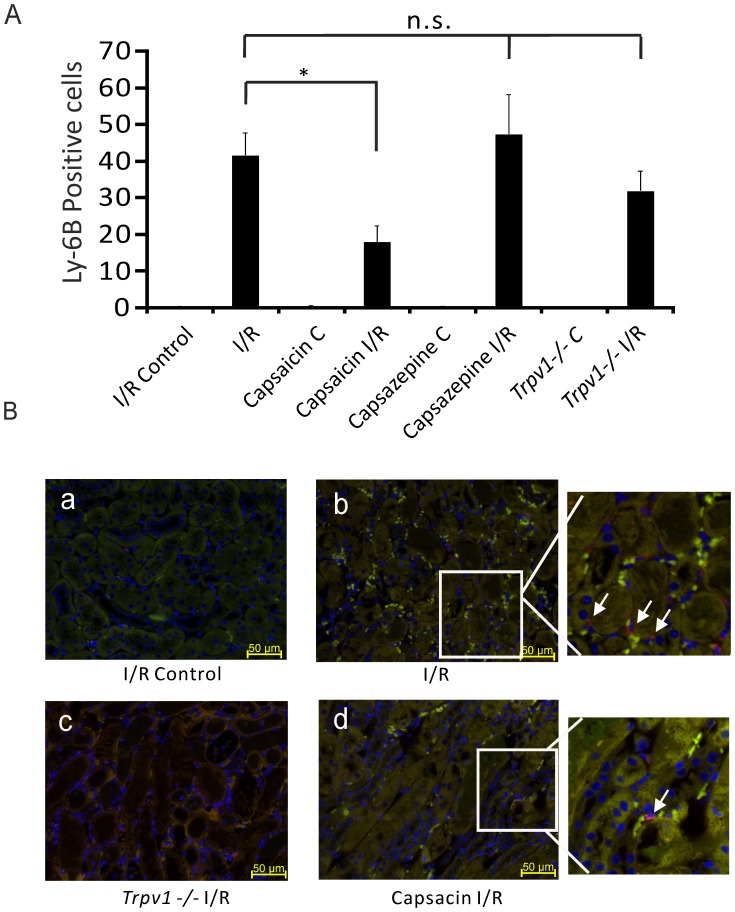
Lower Ly-6B.2 positive cells in the kidneys after I/R-induced AKI in mice treated with capsaicin, but not with capsazepine or in *Trpv1*−/− mice. Ly-6B.2 positive cells were analyzed in the medulla (red color in panels B, marked by arrows). *Panels A:* Ly-6B.2 positive cells per field view in ipsilateral kidneys (I/R Control) and kidneys after I/R (I/R) injury of control mice. Ly-6B.2 positive cells in ipsilateral kidneys (Capsaicin C) and kidneys after I/R injury (Capsaicin I/R) of mice pretreated with capsaicin. Ly-6B.2 positive cells in ipsilateral kidneys (Capsazepine C) and kidneys after I/R injury (Capsazepine I/R) of mice treated with capsazepine. Ly-6B.2 positive cells in ipsilateral kidneys (*Trpv1*−/− C) and kidneys after I/R injury (*Trpv1*−/− I/R) of *Trpv1*−/− mice. *, P<0.05. P>0.05; not significant. **Panels B:** Immunohistological sections. (a) I/R Control, images of Ly-6B.2 positive cells in ipsilateral kidney before renal I/R injury of a control mouse. (b) I/R, Ly-6B.2 positive cells in a kidney after I/R injury of a control mouse. (c) Capsaicin I/R, Ly-6B.2 positive cells in a kidney after I/R injury of a mouse pretreated with capsaicin. (d) Trpv1−/− I/R, Ly-6B.2 positive cells in a kidney after I/R injury of a *Trpv1*−/− mouse. Scale bar 50 µm. Magnification ×400.

Ly-6B.2 is a ∼25–30 kDa GPI-anchored, heavily glycosylated protein expressed on neutrophils, inflammatory monocytes and some activated macrophages. Figure 4 shows that mice treated with capsaicin (0.3 mg/kg) showed less Ly-6B.2 positive cells in kidneys after I/R injury compared to control mice after AKI (Figure 4A, 17.95±4.54 per field view, n = 5 vs 41.62±6.23 per field view, n = 4, respectively, *P*<0.05). Kidneys of mice treated with capsazepine (50 mg/kg, Figure 4A, 47.40±10.7 per field view, n = 5, *P*>0.05) or *Trpv1−/−* mice (Figure 4A, 31.9±5.47, n = 4, *P*>0.05) showed Ly-6B.2 positive cells similar to control mice after I/R injury.

**Figure 5:**
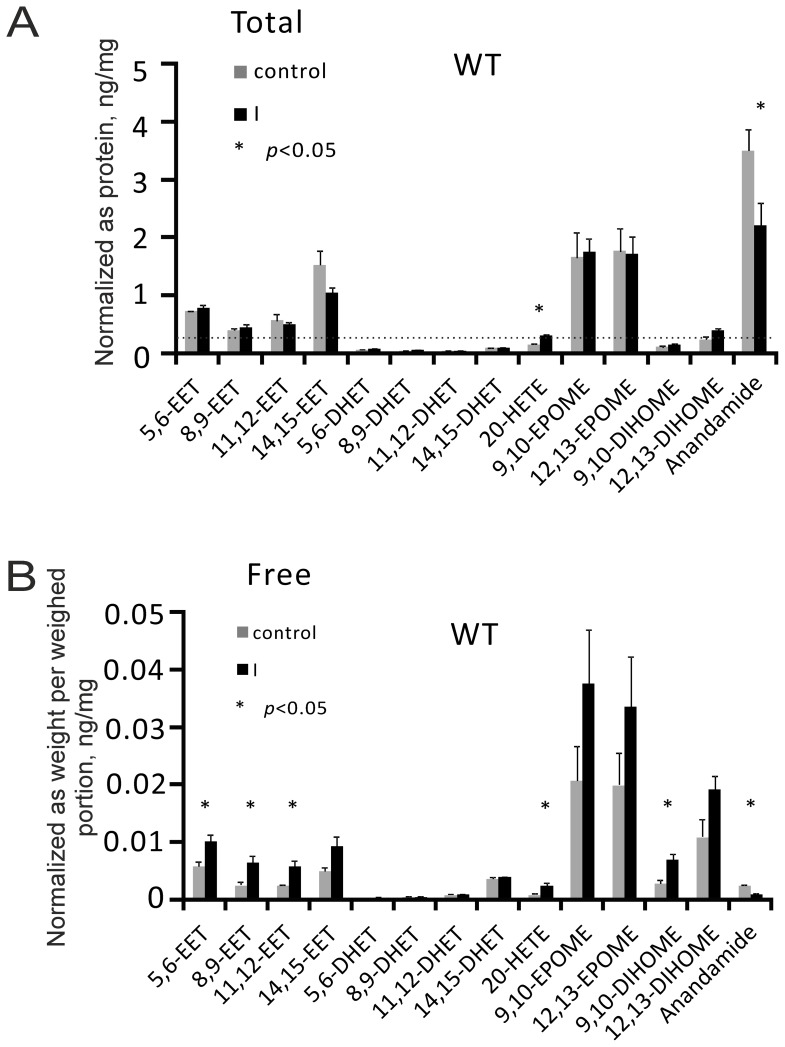
Eicosanoids and anandamide levels in non-ischemic (control) and ischemic (I) kidneys of (WT) wild-type mice after I-induced AKI. 5,6-EET, 5,6-epoxyeicosatrienoic acid; 8,9-EET, 8,9-epoxyeicosatrienoic acid; 11,12-EET, 11,12-epoxyeicosatrienoic acid; 14,15-EET, 14,15-epoxyeicosatrienoic acid; 5,6-DHET, 5,6-dihydroxyeicosastrienoic acid; 8,9-DHET, 8,9-dihydroxyeicosastrienoic acid; 11,12-DHET, 11,12-dihydroxyeicosastrienoic acid; 14,15-DHET, 14,15-dihydroxyeicosastrienoic acid; 20-HETE, 20-hydroxyeicosatetraenoic acid; 9,10-EPOME, 9(10)epoxy-9Z-octadecenoic acid; 12,13-EPOME, 12(13)epoxy-9Z-octadecenoic acid; 9,10-DIHOME, 9(10)-dihydroxy-12Z-octadecenoic acid; 9,10-DIHOME, 9(10)-dihydroxy-12Z-octadecenoic acid. n = 5 in each group.

### Endovanilloids after I-induced AKI

Figure 5A shows that levels of epoxides of arachidonic acid, namely the epoxyeicosatrienoic acid (EET) regioisomers 5,6-EET, 8,9-EET, 11,12-EET, 14,15-EET, were relatively high in non-ischemic kidneys. Similar findings were observed for the 9,10- and 11,12-epoxides of linoleic acid (9,10-EpOME,11,12-EpOME). Figure 5B shows that ischemia does slightly increased release of EETs, 20-HETE, and 9,10-DiHOME. Diols of these metabolites (5,6-DHET, 8,9-DHET, 11,12-DHET, 14,15-DHET, 9,10 DiHOME, 12,13-DiHOME) were present, but at negligibly small levels. Anandamide was present only in bound form at relatively high levels, but decreased during acute ischemia (panel A).

### Endovanilloids after I/R-induced AKI

**Figure 6:**
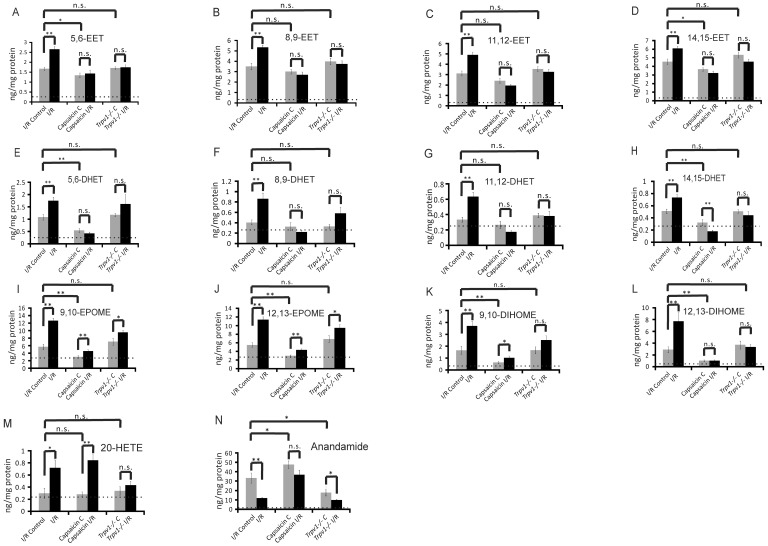
Eicosanoids and anandamide levels in non-ischemic (I/R Control) and ischemic (I/R) kidneys of wild-type mice after I/R-induced AKI, in non-ischemic (Capsaicin C) and ischemic (Capsaicin I/R) kidneys of wild-type mice after I/R-induced AKI, and in non-ischemic (*Trpv1*−/− C) and ischemic (*Trpv1*−/− I/R) kidneys of *Trpv1*−/− mice after I/R-induced AKI. n>8 in each group.

Figure 6 (panels A–L) shows that I/R increased levels of EETs, DHETs, EpOMEs and DiHOMEs in injured kidneys. However, the increase in EETs/DHETs was neither seen in injured kidneys of capsaicin treated mice nor injured kidneys of *Trpv1*−/− mice, which indicates that EETs are unlikely involved in the beneficial effects of capsaicin on outcome of I/R –induced AKI.

Figure 6 (panels I,J) shows that I/R increased levels of both 9,10- and 11,12-epoxides of linoleic acid (9,10-EpOME,11,12-EpOME) in kidneys. TRPV1 deficiency did not affect 9,10-EpOME and 11,12-EpOME levels in non-injured kidneys and in kidneys after I/R injury (panels I,J). In contrast, capsaicin reduced levels of 9,10-EpOME and 11,12-EpOME and their diols (panels K,L) in non-injured kidneys and prevented increases in 9,10-EpOME and 11,12-EpOME in I/R-injured kidneys (panels I,J). Together, the data indicate capsaicin reduces EpOMEs/DiHOMEs in kidneys *in vivo* through activation of TRPV1, an effect that is preserved in kidneys after I/R injury.

Figure 6 (panel M) shows that neither TRPV1 activation by capsaicin nor deficiency of TRPV1 affected levels of 20-HETE in non-injured kidneys. Capsaicin did not affect the increase in 20-HETE in injured kidneys after I/R; nevertheless, TRPV1 channels might be involved in I/R-induced increase in 20-HETE levels since *Trpv1*−/− kidneys showed no increase in 20-HETE.

Of note, TRPV1 activation by capsaicin increased anandamide levels in non-injured kidneys, whereas deficiency of TRPV1 was associated with reduced levels (Figure 6, panel N). Although I/R resulted in decreased levels of anandamide in injured kidneys of wild-type mice, this effect was inhibited by capsaicin but not by TRPV1 deficiency. Together, the data suggest that pharmacological TRPV1 activation by capsaicin can increase levels of anandamide in injured kidneys after I/R.

### Body temperature

There were no differences in core body temperature between the verum groups and the control group during the perioperative procedure (see methods).

## Discussion

In the present study, we showed for the first time that I/R–induced AKI is not affected by inhibition of TRPV1 channels. AKI was assessed by three parameters: by measuring serum creatinine, by scoring tubular damage and by NGAL/Ly-6B.2 immunohistochemistry. Our data are consistent with previous results demonstrating that systemic activation of TRPV1 channels by capsaicin or other agonists mitigates I/R-induced AKI [9] [10] [11]. Our results may have clinical implications for treatment of resistant hypertension by denervation of renal primary sensory nerves in man, i.e. safety issues of this procedure in patients who undergo renal denervation and which is increasingly used in Western countries [28]. There is concern that this procedure may cause harm on the kidney’s ability to respond to ischemic stimuli later in life of these patients [15], in particular since clinical long-term results of patients who underwent this procedure are not available [29].

Ischemic acute renal failure is a frequent clinical syndrome with a high morbidity and mortality [30]. Reperfusion of previously ischemic renal tissue initiates a series of complex cellular events that results in injury and the eventual death of renal cells from a combination of apoptosis and necrosis [31]. Recent pre-clinical data indicate that activators of TRPV1 can improve the outcome of ischemic AKI [9] [10] [11]. Ueda et al. observed that capsaicin (3, 10, and 30 mg/kg orally) 30 minutes before ischemia dose-dependently attenuated I/R-induced renal dysfunction [10]. Mizutani, et. al. suggested that activation of sensory neurons could reduce I/R–induced AKI by attenuating inflammatory responses through enhanced endothelial PGI_2_ production [32]. Kyoko U, et.al. proposed inhibition of inflammatory cells by capsaicin in this response [9]. Our data on Ly-6B.2 immunohistochemistry support this notion. However, it has not been studied whether direct inhibition of these channels is detrimental in AKI or not. In present study, we used a solitary kidney I/R mouse model of AKI to study the effects of pharmacological inhibition of TRPV1 channels (using capsazepine) and genetic ablation of TRPV1 (using *Trpv1−/−* mice). For comparison, we studied the effects of TPRV1 stimulation (using the TRPV1 agonist capsaicin). We found that inhibition of TRPV1 channels does not affect the outcome of I/R-induced renal injury. Three injury parameters demonstrated that TRPV1 inhibition does not cause harm in I/R -induced AKI. Although the relatively high concentration of capsaicin used in this study to activate TRPV1 *in vivo* may have additional effects not related to TRPV1, these three injury parameters demonstrated a beneficial role of capsaicin in I/R-induced AKI as previously reported by other groups for capsaicin and other TRPV1 agonists [9] [10] [11].

Of note, Ueda K, et al. proposed that capsazepine could be an inappropriate drug to study the role of TRPV1 inhibition in I/R-induced renal injury since this drug was not able to antagonize TRPV1 agonist-induced renoprotective effects [9]. As a result, this group did not study directly the effects of capsazepine on I/R-induced AKI. We used a genetic approach (*Trpv1*−/− mice) and pharmacological testing (capsazepine) to study the role TRPV1 inhibition in I/R-induced AKI. Our results demonstrate that AKI is not affected by TRPV1 inhibition. Moreover, tubular damage, NGAL scores and Ly-6B.2 positive cells per field view were not increased in non-ischemic ipsilateral kidneys of capsazepine (50 mg/kg) treated mice (0±0 (n = 10), 0.003±0.002% (n = 10) and 0.22±0.22(n = 5), respectively, *P*>0.05). The latter data demonstrate that capsazepine at 50 mg/kg is not toxic to the mouse kidney itself.

On the other hand, reduction in body temperature has been shown to protect the kidney from ischemic injury [33]. Of note, TRPV1 agonists can cause hypothermia, while TRPV1 antagonists are known to induce hyperthermia in multiple species (rats, dogs, and monkeys), demonstrating that TRPV1 function in thermoregulation [34]. Thus, changes in body temperature in response to capsaicin and/or capsazepine may be involved in effects of these agents on the outcome of AKI. In the present study, surgical procedures were done under body temperature-controlled conditions. Our results demonstrate that changes of body temperature are neither responsible for the protective effects of capsaicin nor for the lack of effects of TRPV1 inhibition on the outcome of I/R-mediated AKI.

In conclusion, pre-ischemic inhibition of TRPV1 in mice does not affect the outcome of I/R-induced renal injury, while activation of these channels mitigates AKI. The mechanisms of the beneficial effects of capsaicin are unknown, but are likely caused via TRPV1-mediated mechanisms in afferent sensory nerves and inhibition of inflammatory responses [9] [10] [11]. Consistently, we found less Ly-6B.2 positive cells in kidneys after I/R of capsaicin treated mice. The beneficial effects of capsaicin may involve release of a substance from TRPV1-expressing nerve endings that exerts a protective action against ischemia/reperfusion-induced kidney injury. Somatostatin is an interesting candidate [35] [36], which can act as autocrine “endovannilloid” to modulate peripheral TRPV1 receptors [37] [38], but so far has not been implicated in AKI. Our measurements of other endovanilloids [19] in kidney tissue demonstrate that anandamide and/or EpOMEs/DiHOMES, but not 20-HETE or EETs, are possible candidates to be involved in the beneficial effects of capsaicin on the outcome of I/R-induced AKI. Although EpOMEs/DiHOMES [20] [23] and anandamide analogs/receptor agonists [39] have been recently suggested to represent novel therapeutic targets in AKI, our data suggest that future studies on their role in AKI should consider pathways of TRPV1 signaling to understand their molecular actions.

Our data suggest that lack of functional TRPV1 channels does not cause harm to the kidney’s ability to respond to support ischemic stimuli. These data may have important implications for interpretation of data on long-term safety of RDN in man. Since this procedure impairs responsiveness of TRPV1-rich renal primary sensory nerves, our data will contribute to the validity of arguments aimed to address the question whether this procedure is effective and safe to treat resistant hypertension or even to do nothing, than to risk causing more harm than good. Moreover, discovery of novel pharmacological TRPV1 modulators may be a successful strategy for better treatment of acute kidney failure.
