# Correction: Role of TRPV1 Channels in Ischemia/Reperfusion-Induced Acute Kidney Injury

**DOI:** 10.1371/journal.pone.0115469

**Published:** 2014-12-09

**Authors:** The PLOS ONE Staff

The Acknowledgments section of the published article contains errors. Please view the correct Acknowledgments here:

We thank Joon-Keun Park (Hannover Medical School, Hannover, Germany) for performing analytical analyses of serum creatinine levels. We thank Marwan Mannaa, Gabriele N'diaye, May-Britt Köhler and Petra Berkefeld for expert technical advice and help in experiments.
